# Bacterial Adaptive Memory in Methicillin-Resistant *Staphylococcus aureus* from Endotracheal Tubes

**DOI:** 10.3390/pathogens13020144

**Published:** 2024-02-05

**Authors:** Laia Fernández-Barat, Ruben López-Aladid, Nil Vázquez, Roberto Cabrera, Jordi Vila, Miquel Ferrer, Antoni Torres

**Affiliations:** 1 Centro de Investigación Biomedica En Red-Enfermedades Respiratorias (CibeRes, CB06/06/0028) and Institut d’Investigacions Biomèdiques August Pi i Sunyer (IDIBAPS), 08036 Barcelona, Spain; rlopeza@recerca.clinic.cat (R.L.-A.); nvazquez@recerca.clinic.cat (N.V.); rcabrera@recerca.clinic.cat (R.C.); mferrer@clinic.cat (M.F.); 2 University of Barcelona, 08193 Barcelona, Spain; jvila@clinic.cat; 3 Microbiology Service at Hospital Clinic and Institute of Global Health (ISGlobal), 08036 Barcelona, Spain; 4 Pulmonary and Critical Care Unit, Respiratory Institute, Hospital Clinic, 08036 Barcelona, Spain

**Keywords:** methicillin-resistant *Staphylococcus aureus*, biofilm, endotracheal tubes, nosocomial pneumonia, ventilator-associated pneumonia, gene expression

## Abstract

Objectives: To evaluate the expression dynamics of biofilm genes in methicillin-resistant *Staphylococcus aureus* (MRSA) retrieved from endotracheal tubes (ETT) and to determine how gene regulation is attenuated in vitro where host–environmental factors are no longer present. Methods: Biofilm was grown (24 h) in tryptic broth soy plus 0.25% glucose for a clinical MRSA isolate in planktonic state and after sessile growth named ETT-MRSA (S2, S3, S4, S5, S6, S7). Gene expression of five biofilm-related genes (*icaC*, *clfB*, *ebps*, *fnbB*, and *RNA III*) was assessed consecutively from day 1 to day 4 after ETT growth through real-time PCR. 16S rRNA was used as a control. Results: The MRSA isolates retrieved from ETT were capable of producing biofilms dependent on *ica*. The gene expression dynamics of ETT-MRSA changed progressively compared to planktonic MRSA gene expression under both ambient air (*p* < 0.001) and ambient air with 5% CO2 (*p* < 0.001). Dynamic assessment of *icaC* expression in both atmospheric conditions showed progressive downregulation in vitro compared to in vivo ETT biofilms. The expression patterns of *clfB* and *ebps* genes were similar to *icaC.* In contrast, the expression of the *RNA III* gene showed progressive upregulation from day 1 to day 4 (*p* < 0.001). Conclusions: MRSA loses its biofilm gene expression in vitro, by adaptive features across multiple generations, as evidenced by the progressive downregulation of *icaC* and upregulation of *RNA III*. These findings underscore the significance of host–environment dependence in regulating bacterial biofilm genes, highlighting its importance in diagnostics. Bacterial strains lose their host-specific characteristics as they are cultured in vitro.

## 1. Introduction

In an intensive care unit (ICU) the presence of an endotracheal tube (ETT) in ventilated patients impairs mucociliary clearance, disrupts the natural mechanisms of defense for mucus clearance, and causes airway microbial dysbiosis. The ETT promotes the accumulation of colonized tracheobronchial secretions and the formation of bacterial biofilms that can easily translocate into lower airways and increase the risk of ventilator-associated pneumonia (VAP) or its relapse [1,2,3]. *Staphylococcus aureus* is among the most important causes of VAP, and especially MRSA is associated with increased treatment failure, higher cost, worse prognosis, and higher mortality than methicillin-sensitive *S. aureus* (MSSA) in VAP [4].

Bacteria can be in vitro characterized as strong or weak biofilm producers by a simple microtiter plate assay [5] or by the biofilm ring test, among other methods [6]. However, it is not always straightforward to extrapolate these in vitro results to bacterial behavior in clinical settings [7]. Additionally, the lack of a standardized method to determine biofilm capability hinders comparisons among results. The usefulness of incorporating biofilm capability into microbial diagnostics is currently a topic of debate. Not only can individual bacteria in biofilms exhibit different susceptibility patterns than planktonic bacteria, but they can also become highly tolerant to antibiotics when growing in biofilm aggregates [8]. To address this gap, a new tool called the Antibiofilmogram^®^ has been developed to determine the biofilm minimum inhibitory concentration (bMIC) [9]. However, bacterial biofilm capability appears to be a plastic trait rather than a constitutive one. This fundamental issue poses a significant challenge for clinical researchers studying biofilm-associated infections. Biofilm production in MRSA has been attributed to the production of exopolysaccharide, polysaccharide intercellular adhesion (PIA), or polymeric N-acetyl-glucosamine (PNAG), synthesized and exported by proteins encoded by the *icaADBC* genes. However, *S. aureus ica* independent biofilm producers have also been described. In these, microbial surface components that recognize adhesive matrix molecules (MSCRAMMs) rather than PIA/PNAG play an important role [10,11]. These MSCRAMMs can bind to one or more host extracellular matrix factors including elastin (*ebpS*), fibronectin B (*fnbB*), bacterial ligands clumping factors B (*clfB*), and fibrinogen [12]. These proteins share a common signal sequence for secretion as well as for anchoring to the cell wall.

In contrast, *RNAIII*, the effector of the *agr* quorum-sensing system, plays a key role in virulence gene regulation in *S. aureus*. *RNAIII* affects MRSA and MSSA genes through another important *S. aureus* global transcriptional regulator, *MgrA*, which has been shown to affect more than 350 genes involved in virulence, antibiotic resistance, autolysis, and biofilm formation [13].

Previous studies suggested that MRSA increased biofilm production capability after growing within ETT in a reversible way [14]. In addition, recent investigations have suggested that surface-sensing signals can be propagated as a type of memory across multiple bacterial generations. The ability to memorize and adjust current behaviors can lead to efficient adaptation to the recurring host environment [15]. Subsequently, our aim was to perform a dynamic assessment of the expression of biofilm-related genes of ETT-MRSA compared to MRSA in planktonic growth to determine the existence of bacterial adaptive features to nosocomial settings at a gene expression level. Finally, we also assessed how bacterial gene expression responds to different atmospheric growth conditions as it occurs during mechanical ventilation.

## 2. Materials and Methods

### 2.1. The Pig Model of MRSA Pneumonia

The model of large Landrace white pigs with MRSA-VAP was previously validated as a useful model to test MRSA therapies, including pharmacokinetics and pharmacodynamics, histopathology, clinical outcomes as well as bacterial burden [1]. Pigs were intrabronchially challenged with 75 mL inoculum with 10^6^ colonies forming units (CFU) per mL of a clinical MRSA strain in exponential growth phase (OD_600_~1–5). At 12 h after MRSA challenge, 6 animals were randomized to be treated with placebo (n = 3, saline 0.9%) or vancomycin (n = 3, 15 mg∙kg^−1^ every 12 h IV). At 84 h of mechanical ventilation, animals were sacrificed and all the ETTs were systematically kept frozen at −80 °C until analysis.

Thus, the planktonic and all the ETT-MRSA isolates included in this study belonged to the same PVL-negative MRSA clone (ST125) and agr II, obtained from an ICU patient. Methods such as typing, pulsed-field gel electrophoresis (PFGE), the microtiter plate assay, or biofilm thickness were previously reported [16,17].

### 2.2. Bacterial Strains and Growth Conditions

The in vitro ability of all isolated MRSA strains to form biofilm was assessed by microtiter plate assay slightly modified as follows. All isolates (MRSA not previously grown in ETT, named “MRSA in”, and ETT-MRSA, isolated from ETT from piglets mechanically ventilated (69 ± 16 h), were first cultured overnight at 37 °C with agitation on tryptic soy broth media (TSB; SIGMA-ALDRICH, Spain). After this, each isolate was diluted 1/50 in 200 μL TSB with 0.25% glucose in a microwell of a 96 flat bottom microtiter plate (polystyrene, sterile; SIGMA-ALDRICH, Barcelona, Spain) and incubated, without shaking, overnight at 37 °C. Then, all non-attached bacteria were removed, 100 μL of PBS (Roche, Barcelona, Spain) was added, and 5 min of sonication at 40 kHz was applied in order to disaggregate the biofilms and to perform the RNA extraction.

**Figure 1:**
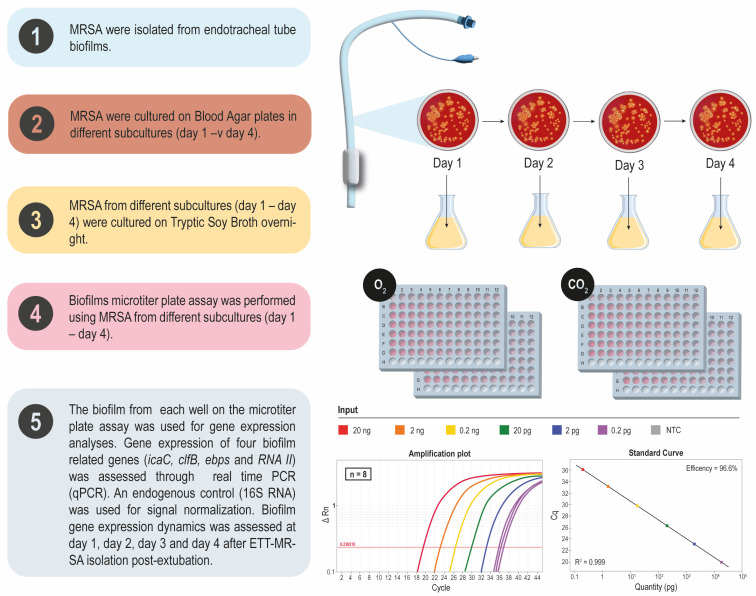
The planktonic MRSA and the 6 ETT-MRSA isolates were unfrozen on blood agar plates from 1 to 4 consecutive culture transfers, representing the dynamics post-extubation. After an overnight saturated culture on liquid tryptic broth soy (TSB), a biofilm on a microtiter plate was grown in 24 h (TSB + 0.25% glucose) for each isolate. Non-attached bacteria were discarded. Biofilm-grown bacteria were sonicated in 100 µL PBS. Then, 75 µL of each well was incubated with an appropriate volume of RNAprotect (QIAGEN, Barcelona, Spain). The remaining 25 µL of each well was used for counting colony-forming units (CFU). The experiment was performed using 3 intra- and 3 inter-assay replicates for each MRSA isolate.

This assay was performed in MRSAin and each ETT-MRSA isolate from 1 to 4 consecutive subcultures in blood agar plates that corresponded to day 1, day 2, day 3, and day 4 after ETT-MRSA isolation post-extubation, respectively (Figure 1). Ultimately, two different atmosphere growth conditions were compared (ambient air (O_2_) or ambient air supplemented with 5% CO_2_ (CO_2_)). Intra- and inter-assay triplicates were performed. 

### 2.3. cDNA Synthesis

cDNA was synthesized from mRNA using Primer Script RT Reagent kit according to manufacturer instructions (Takara Bio Inc., Kusatsu, Japan). The same amount of total RNA (500 ng/10 µL) was reverse transcribed in reaction volume of 10 µL. RNA quality determination: The concentration and purity of the total RNA were spectrometrically determined using a NanoDrop 1000™ (Thermo Scientific, Waltham, MA, USA). Three independent measurements of the same sample were performed. The absorbance ratio A260/A280 was used as an indicator of protein contamination and A260/A230 as an indicator of polysaccharide, phenol, and/or chaotropic salt contamination. RNA was stored at −80 °C.

### 2.4. Gene Expression Quantification

Biofilm was grown (24 h) in tryptic broth soy plus 0.25% glucose for a clinical MRSA isolate in planktonic state and after sessile growth named ETT-MRSA (S2, S3, S4, S5, S6, S7). Gene expression of five biofilm-related genes (*icaC*, *clB*, *ebps*, *fnb,* and *RNA III*) was assessed through real-time PCR (qPCR). An endogenous control (16S rRNA) was used for signal normalization. Biofilm gene expression dynamics were assessed at day 1, day 2, day 3, and day 4 of subculture after ETT-MRSA isolation.

Oligonucleotide primers for the detection of 16S rRNA, *icaA*, *fnB*, *clb*, *ebps,* and *RNA* III were previously described (Table 1). qPCR analysis was performed using commercial qPCR master mix: Sybr Premix Ex Taq Reagent kit according to manufacturer instructions (Takara Bio Inc., Kusatsu, Japan) [18].

The total reaction volume of 20 µL reactions contains 2 µL diluted cDNA, 2µL of each primer at 3 µM concentration, 4 µL nuclease-free H_2_O, and 10 µL of the respective 2× master mix. PCR optimization and efficiency were based on planktonic MRSA strain. Primer efficiencies were determined by the dilution 10× method as well as performing a temperature gradient reaction from 50 to 65 °C. At 60 °C, set of primers had the best and most similar efficiency values. qPCR run was performed on a Light Cycler 96 (Roche, Risch-Rotkreuz, Switzerland) with the following cycle parameters: 94 °C for 30 s, 40 cycles of 94 °C for 5 s, 60 °C for 30 s, and 68 °C for 15 s. qPCR products were analyzed by melting curves for unspecific products or primer dimer formation. Relative fold increase in specific mRNA transcripts in biofilms compared with planktonic cultures was calculated using 2ΔCt method based on a mathematical model where 2 stands for the 100% reaction efficiency and ΔCt = Ct (housekeeping gene) − Ct (target gene).

### 2.5. RNA Extraction DNase Treatment Protocols

Previously, 75 µL of bacteria were suspended in 500 µL RNA protect storage at −80 °C. To disrupt bacterial cells, we used enzymatic lysis and spin column extraction by RNAminieasy kit (QIAGen, Heidelberg, Germany). Total RNA was isolated according to the manufacturer’s instructions, with the following optimization: the appropriate cell lysis was performed using 10 mg/mL of lysozyme and 5 mg/mL of lysostaphin (Sigma, St Louis, MO, USA) for 60 min at 37 °C in a shaker at 120 rpm [1]. Afterward, samples were centrifuged at 14,000× *g* for 10 min and supernatants were transferred to a new tube and mixed with equal volume of 100% ethanol. The samples were transferred to the QIAgen spin columns according to the manufacturer’s instructions. All steps were conducted at room temperature and RNA was digested with DNase I (Invitrogen, Carlsbad, CA, USA). Briefly, 2 µL of DNase I and 5 µL of reaction buffer were added to the RNA sample and incubated at 37 °C for 30 min. Then, to inactivate the DNase I enzyme, 5 µL of 30 mM EDTA was added to the mixture and incubated at 65 °C for 10 min [18]. 

### 2.6. Statistical Analyses

Data are reported as the median (interquartile range, IQR) or as mean ± SD, and were tested for normal distribution using the Shapiro–Wilk test. Qualitative or categorical variables were compared between groups with the Mann–Whitney test. Paired variables were assessed using the non-parametric Wilcoxon signed ranks test and independent samples were assessed using the non-parametric Kruskal–Wallis test. The Bonferroni correction was used for all post hoc comparisons. To determine the relationship between quantitative variables, the Spearman rank-order correlation coefficient was used. A two-sided *p*-value < 0.05 was considered statistically significant. All statistical analyses were performed using IBM SPSS, Version 21 software (IBM SPSS statistics, 21, Chicago, IL, USA).

## 3. Results

**Figure 2:**
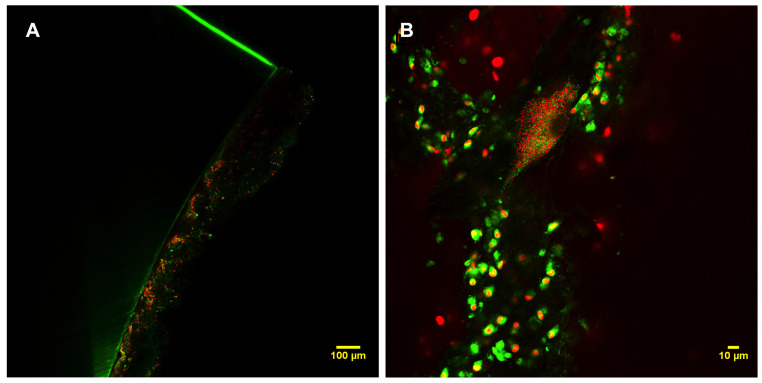
Confocal images of endotracheal tube evidencing MRSA biofilm from a pig with MRSA pneumonia. (**A**) Low magnification and (**B**) high magnification. Live bacterial communities stained with SYTO^®^ 9 emit green light, whereas dead bacteria stained with propidium iodide emit red light. The nucleus of eukaryotic cells, i.e., polymorphonuclear neutrophils, is typically red, while the cytoplasm is green.

MRSA was found in all included ETTs without significant differences regarding MRSA counts obtained from placebo versus vancomycin-treated groups (4.20 (2.74–4.82) vs. 2.42 (1.50–5.46), *p* = 0.69, respectively), or regarding hours of mechanical ventilation (Figure 2).

**Figure 3:**
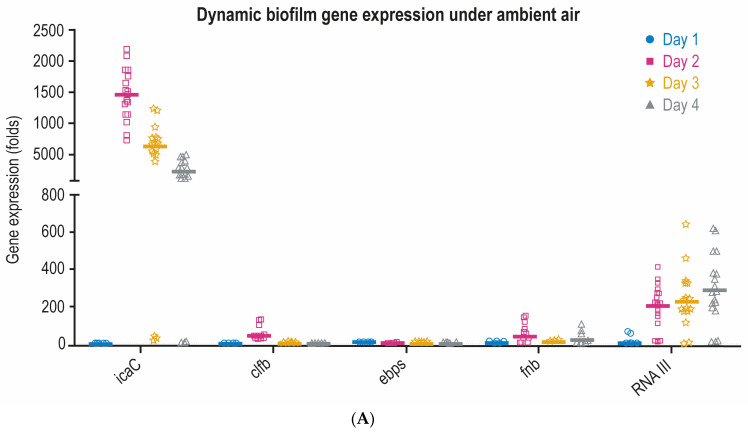

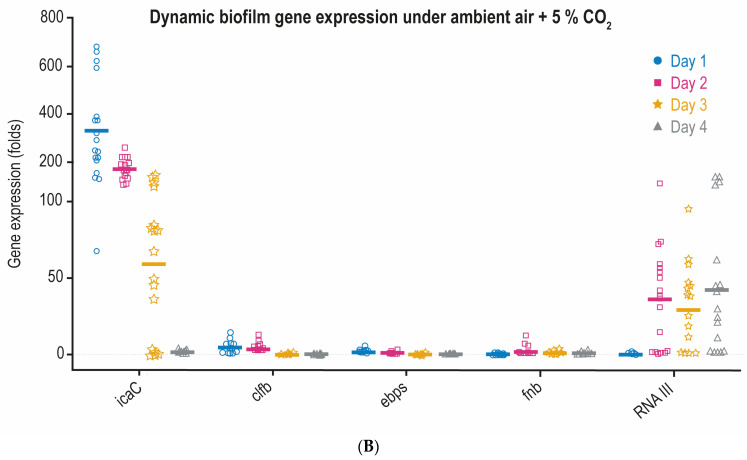
Dynamic biofilm gene expression under ambient air (**A**) and under ambient air supplemented with 5% CO_2_ (**B**).

Gene expression results evidenced our ETT-MRSA isolates were *IcaC*-dependent biofilm producers since the highest levels of gene expression were allocated in the *IcaC* gene in comparison to other genes analyzed (*clb*, *ebps*, *fnb,* and RNAIII) [11]. Gene expression dynamics of the ETT-MRSA (S2, S3, S4, S5, S6, and S7), always with respect to planktonic MRSA, evidenced differences in the expression of all biofilm genes (*icaC*, *clb*, *ebps*, *fnb,* and *RNAIII*) among the different days of consecutive subcultures and under both ambient air and ambient air + 5%CO_2_ (Figure 3A,B). Gene expression dynamics showed progressive downregulation for all genes, but *RNA III* was upregulated.

Specifically, *icaC* expression (folds) drastically downregulated from day 2 to day 4 in ambient air, and from day 1 to day 4 under ambient air + 5%CO_2_. The expression dynamics of *clb* and *ebps* showed a similar pattern to *icaC* though at a lower scale of expression. Dynamics of *Fnb* expression did not present significant differences under ambient air but slight differences under ambient air + 5%CO_2_. Ultimately, *RNAIII* showed an opposite pattern to *icaC* of expression upregulating from day 1 to day 4 under both atmospheric conditions (Table 2), Figures S1–S3.

The effect of atmosphere growth conditions showed that from day 2 to 4 of the subculture gene expression of the five genes was always higher under ambient air compared to ambient air + 5%CO_2_. In spite of a similar dynamic pattern under both atmospheric growth conditions, at day 1 *IcaC* and *clb* expression was higher (*p* < 0.0001) under ambient air + 5%CO_2_ than in ambient air. Results on MRSA burden on the microtiter plate assay did not present significant differences between days of subculture nor atmospheric conditions and are reported in Supplementary Materials Table S1, Figure S4.

MRSA strains S2, S6, and S7 were isolated from the ETTs of vancomycin-treated pigs whilst S3, S4, and S5 were isolated from control-treated pigs. No significant differences were detected for Ica, clb, ebps, fnb, RNAIII expression on D1, D2, D3, and D4 post-extubation comparing isolates exposed to control and vancomycin systemic treatments during intubation neither under atmospheric conditions nor at atmospheric conditions + CO_2_, Figure S4.

## 4. Discussion

Our study demonstrated that MRSA biofilm gene expression in nosocomial settings is a dynamic and adaptable process that is influenced by environmental factors. We observed that biofilm gene expression in MRSA retrieved from endotracheal tubes was retained across multiple generations as a type of adaptive memory until disappearing after four consecutive in vitro subcultures where the ETT stimulus and host factors were absent. Furthermore, our investigation suggests that biofilm genetic regulation is sensitive to atmospheric growth conditions, indicating that different ventilator settings in the ICU may contribute to ETT biofilm formation. These findings highlight the importance of bacterial gene expression as a reporter of the host environment and underscore the potential of these adaptive features as a diagnostic tool for biofilm infections, which is currently underestimated.

The “immunological memory” of eukaryotic cells, which persists even after cells are removed from the host, has long been raised as an important adaptive trait to rapidly and effectively respond to re-infections [19]. Indeed, it has been elucidated that regulatory T cells also have mechanisms to transiently regulate their intrinsic memory in order to avoid a state of perpetual immunosuppression. Regarding prokaryotic cells, the *Bacillus subtilis* motility state, for instance, is an individual state, in which cells are insensitive to how long they remain motile. In contrast, growing in bacterial chains, the earlier steps of multicellularity, requires some degree of memory that enables cell-to-cell cooperation, for such a long-term commitment as a biofilm [19]. Additionally, *E. coli* behavior in a microfluidic device differentiates phenotypic from hysteretic memory. The first makes reference to the transmission of stable intracellular proteins among multiple generations that allow bacterial adaptation to fluctuating environments. The second enhances adaptation when environments fluctuate even more rapidly. The combined use of both biological memory systems avoids unnecessary fitness costs for bacteria [20].

In *P. aeruginosa,* coupled oscillations of cAMP levels and type IV pili activity serve as a memoristic mechanism to adaptively adhere to surfaces. Thus, “surface-sentient” bacteria, which have previously landed on a surface, express at time “0” higher levels of cAMP and type IV pili, which is ultimately linked to the c-di-GPM increase, a determinant in early adhesion steps [21]. The ETT-MRSA strain demonstrated significantly higher expression of the *icaC*, *clb*, and *ebps* genes compared to the identical MRSA clone in its planktonic state before exposure to the ETT. However, the elevated gene expression related to biofilm formation in ETT-MRSA was diminished after four consecutive days of subcultures. Thus, our ETT-MRSA isolates were *IcaC*-dependent biofilm producers. Our finding interestingly suggests that the decreased icaADBC operon expression linked to the acquisition of the gene *mecA*, previously described, can be reverted in vivo. Indeed, the biofilm gene expression found in the present study is in line with our previous results in which we observed that biofilm production dynamics followed the same pattern of ex vivo downregulation [14].

The print of the host’s environment on bacterial behavior is a determinant in microbiology diagnostics. With a similar approach as ours, Kordes et al. demonstrated that *P. aeruginosa* virulence can be increased in a low virulent isolate upon serial passages in a *Galleria mellonella* infection model [15]. However, this virulent evolved strain reverted to the original non-virulent after 4 days of rich LB growth [15]. RNA III encodes δ- toxin that lyses eukaryotic host cells but it also acts as an important transcriptional regulator of *agr,* which targets key virulence factors such as proteases and toxins, some of them involved in the biofilm disassembly [22]. The agr quorum sensing system switches on or off under a fine regulation control, which allows bacteria to start the biofilm mode of growth and persist under stressful environmental conditions or to start the virulent planktonic dispersal mode colonizing or invading new sites when, for instance, the biofilm is at an advanced stage [23,24]. In our study, this inverse coupled regulation between biofilm and virulence genes is well exemplified through the opposite gene expression that follows *IcaC* and RNAIII. Indeed, highly virulent *P. aeruginosa* strains were also associated with a lower gene expression of biofilm-related genes [15]. Therefore, the utility of quorum-sensing signal molecules as new targets for therapeutic intervention is a rising matter of interest.

The virulent evolved strain of Kordes et al. was not linked to genetic variation and was also reproduced in media supplemented with excess linolenic acid, an environmental condition that prevails in *G. mellonella* [15]. With a similar attempt, we aimed to demonstrate how the different air composition environments present in intubated ICU patients may condition ETT biofilm dynamic gene expression. Interestingly, and in line with our previous results [14], the lower gene expression under ambient air on day 1 subculture was attributed to higher metabolic rearrangement required from the ETT to this in vitro condition. In contrast, such rearrangement did not occur under ambient air + 5% CO_2_ since the partial pressure of CO_2_ in such a condition is very similar (38 mmHg) to that found in normal airways (35–45 mmHg). However, from day 2 to 4, ETT-MRSA biofilm gene expression was lower under ambient air + 5%CO_2_, which is in agreement with the microtiter plate assays [14]. Other studies reported discrepancies regarding biofilm production under CO_2_-rich conditions, but, moreover, they used different methods, some differed with or lacked [11] the dynamic biofilm assessment and the comparison between biofilm vs. its planktonic bacterial counterpart, which allows for the deepest inspection of bacterial biofilm regulation under different atmospheric environments [25,26,27,28].

Several limitations deserve to be mentioned. First, MRSA strains were kept frozen after extubation. However, the same thawing was applied to each MRSA isolate, thereby eliminating any potential bias. Second, the ETT-MRSA was obtained from a pig model MRSA-VAP, and by pulsed-field gel electrophoresis they were confirmed to belong to the same MRSA clone with whom the pigs were challenged, which in turn was a clinical MRSA isolated from an ICU patient. Ultimately, our experiment was only performed in several ETT-MRSA isolates, but obtained after 4 days of mechanical ventilation, and then reproduced in a real ICU setting.

Our results represent the first in vivo demonstration of an underestimated time dimension effect regarding host environment dependence in bacterial gene expression with respect to their ability to produce biofilms. Our findings, based on MRSA, together with previous findings on other nosocomial pathogens [14,15,22,23] that are consistent with our results, emphasize the underestimated value of bacterial gene expression in diagnostic microbiology. However, researchers interested in pursuing this field of study must consider the host-dependency of biofilm gene expression. Results may vary depending on the in vitro exposure of bacterial strains, which can mask the effect of the host environment on bacterial gene expression.

## Figures and Tables

**Table 1:** Sequences of oligonucleotide primers used for qPCR.

Genes	Nucleotide Sequence of Primers (5′-30)	Accession Numbers	Annealing Temperature	Size (Bp)
*icaC* (intercellular adhesion gene)	5-CTTGGGTATTTGCACGCATT-3	AF086783	60	209
	5-GCAATATCATGCCGACACCT-3			
*fnbB* (fibronectin-binding protein B)	5-ACGCTCAAGGCGACGGCAAAG-3	X62992.1	60	197
	5-ACCTTCTGCATGACCTTCTGCACCT-3			
*clfB* (clumping factor B)	5-AACTCCAGGGCCGCCGGTTG-3	X62992.1	60	159
	5-CCTGAGTCGCTGTCTGAGCCTGAG-3			
*ebps* (elastin-binding protein)	5-GGTGCAGCTGGTGCAATGGGTGT-3	U48826.2	60	191
	5-GCTGCGCCTCCAGCCAAACCT-3			
*RNA III* (virulence factor)	5-GAATTTGTTCACTGTGTCGATAATC-3	HF937103.1	60	114
	5-GAAGGAGTGATTTCAATGGCAC-3			
*16S rRNA* (housekeeping gene)	5-GGGACCCGCACAAGCGGTGG-3	L37597.1	60	191
	5-GGGTTGCGCTCGTTGCGGGA-3			

**Table 2:** ETT-MRSA fold change in mRNA levels of adhesion and biofilm target genes at different days of subculture post-extubation.

	Gene	Type	ETT-MRSA Gene Expression				
			D1 (a)	D2 (b)	D3 (c)	D4 (d)	*p*-Value
Ambient Air	*16s*	REF	1	1	1	1	
	*icaC*	TRG	0.13 (0.03–0.64) ^b,c,d^	14,664.76 (11638.44–17611.76) ^c,d^	6635.01 (4739.38–7799.29) ^d^	2168.50 (1164.38–3676.71)	<0.001
	*clfb*	TRG	0.14 (0.05–0.60) ^b^	33.12 (16.75–43.50) ^c,d^	0.32 (0.27–0.45)	0.65 (0.00–2.23)	<0.001
	*ebps*	TRG	2.88 (0.08–6.16)	3.02 (2.02–4.19) ^c,d^	0.89 (0.33–1.97) ^d^	0.15 (0.10–0.19)	<0.001
	*fnb*	TRG	0.68 (0.00–3.99) ^d^	6.66 (4.50–60.72)	4.36 (0.76–10.93)	6.67 (0.29–14.81)	0.18
	*RNA III*	TRG	0.12 (0.01–0.67) ^b,c,d^	214.76 (137.51–269.44) ^d^	214.83 (157.11–320.93)	275.48 (181.89–404.95)	<0.001
Ambient Air + 5% CO_2_	*16s*	REF	1	1	1	1	
	*icaC*	TRG	270.77 (195.25–439.99) ^b,c,d^	170.27 (132.93–202.17) ^c,d^	58.63 (3.21–91.37) ^d^	1.46 (0.71–2.91)	<0.001
	*clfb*	TRG	3.86 (1.95–7.15) ^b,c,d^	2.37 (1.76–4.59) ^c,d^	0.01 (0.007–0.02) ^d^	0.25 (0.19–0.33)	<0.001
	*ebps*	TRG	1.07 (0.32–2.23) ^c,d^	0.98 (0.34–1.39) ^d^	0.05 (0.032–0.08) ^d^	0.25 (0.06–0.32)	<0.001
	*fnb*	TRG	0.25 (0.08–0.49) ^c^	0.80 (0.55–0.89)	0.98 (0.22–1.82) ^d^	0.56 (0.39–0.85)	0.002
	*RNA III*	TRG	0.05 (0.03–0.13) ^b,c,d^	37.83 (1.76–57.29) ^c,d^	27.36 (1.10–45.19) ^d^	26.27 (1.57–71.72)	<0.001

REF: Reference housekeeping gene, TRG: target gene, D1–4: days. Values expressed in folds (median (IQR)) with respect to MRSAin. Pairwise comparisons ambient air: icaC, all *p* < 0.001. clfb, a,b; b,c; b,d: *p* < 0.001 a,c: *p* = 0.446; a,d: *p* = 0.248; c,d: *p* = 0.078. ebps, a,b: *p* = 0.472; a,c: *p* = 0.012; a,d: *p* = 0.005; b,c; b,d and d,c: *p* < 0.001. RNAIII, a,b; a,c; and a,d: *p* ≤ 0.001; b,c: *p* = 0,528; b,d: *p* = 0.004; c,d: *p* = 0.048. Pairwise comparisons ambient air + 5% CO_2_: icaC, all *p* < 0.001 but a,b: *p* = 0.003. clfb, all *p* < 0.001 but a,b: *p* = 0.133. ebps, a,b: *p* = 0.616; a,c: *p* < 0.001; a,d: *p* = 0.002; b,c and b,d: *p* < 0.001; d,c: *p* = 0.004. fnb, a,b: *p* = 0.020; a,c: *p* = 0.004; a,d: *p* = 0.184; b,c: *p* = 0.586; b,d: *p* = 0.018; d,c: *p* = 0.006. RNAIII, a,b; a,c; a,d: *p* < 0.001 b,c: *p* = 0.215; b,d: *p* = 0.983; c,d *p* = 0.007.

## Data Availability

All data generated or analyzed during this study are included in this published article.

## Supplementary Materials

The following supporting information can be downloaded at: https://www.mdpi.com/article/10.3390/pathogens13020144/s1.
