# Supplementary material for: Bacterial Adaptive Memory in Methicillin-Resistant Staphylococcus aureus from Endotracheal Tubes
Source: Pathogens. 2024 Feb 5;13(2):144. doi: 10.3390/pathogens13020144 (PMC10892081; doi:10.3390/pathogens13020144)
Supplement: Supplementary file 1 [file pathogens-13-00144-s001.zip › pathogens-2807131-supplementary.pdf]

Supplementary material

Bacterial Adaptive Memory in Methicillin-Resistant *Staphylococcus aureus* from Endotracheal Tubes

Laia Fernández-Barat [1,2,*], Ruben López-Aladid [1,2], Nil Vázquez [1,2], Roberto Cabrera [1,2], Jordi Vila [2,3], Miquel Ferrer [1,2,4] and Antoni Torres [1,2,4,*]

[1] Centro de Investigación Biomedica En Red-Enfermedades Respiratorias (CibeRes, CB06/06/0028) and Institut d'Investigacions Biomèdiques August Pi i Sunyer (IDIBAPS), 08036 Barcelona, Spain; rlopeza@recerca.clinic.cat (R.L.-A.); nvazquez@recerca.clinic.cat (N.V.); mferrer@clinic.cat (M.F.)

[2] University of Barcelona, 08193 Barcelona, Spain; jvila@clinic.cat

[3] Microbiology Service at Hospital Clinic and Institute of Global Health (ISGlobal), 08036 Barcelona, Spain

[4] Pulmonary and Critical Care Unit, Respiratory Institute, Hospital Clinic, 08036 Barcelona, Spain

* Correspondence: lfernan1@recerca.clinic.cat (L.F.-B.); atorres@clinic.cat (A.T.)

Table S1. MRSA burden, vancomycin minimum inhibitory concentration and hours of ventilation

			log MRSA UFC/ml	MIC Vancomycin	Hours ventilation
Treatment	Control	1	4.20	1.50	58.00
		2	4.48	1.50	58.00
		3	3.30	1.50	72.00
		4	5.16	1.50	72.00
		5	0.00	.	67.00
		6	2.18	1.25	67.00
	Vanco	1	2.00	1.00	66.00
		2	1.00	1.50	66.00
		3	5.08	1.50	84.00
		4	5.85	1.50	84.00
		5	2.41	1.25	84.00

Figure S1.

MRSA biofilm growth (LogCFU/mL) was registered in each day (1-4) post extubation and under both atmospheric conditions. All these results have been included in the supplementary material. Please find below the main results of this analysis, no significant differences were found between MRSA biofilm growth in CFU comparing day1-day4 between growth in ambient air (1) or ambient air+CO_2 (2) as you can see below:

Percentiles

		O2(1)_CO2(2)	Percentiles 5	10	25	50	75	90	95
Weighted Average (Definition 1)	MeanBDay1	1	8.6898	8.6898	9.2552	10.1834	10.9003	.	.
		2	9.2416	9.2416	9.4546	10.1500	10.5647	.	.
	MeanBDay2	1	10.0000	10.0000	10.0548	10.7996	11.0201	.	.
		2	10.0485	10.0485	10.2742	10.5779	11.1197	.	.
	MeanBDay3	1	10.4225	10.4225	10.6480	10.8838	11.4045	.	.
		2	10.7870	10.7870	10.8594	11.0421	11.1094	.	.
	MeanBDay4	1	8.8580	8.8580	9.7129	10.1228	11.2517	.	.
		2	9.3514	9.3514	9.5051	9.9895	10.6503	.	.
Tukey's Hinges	MeanBDay1	1			9.4437	10.1834	10.7959		
		2			9.5256	10.1500	10.4454		
	MeanBDay2	1			10.0731	10.7996	10.9404		
		2			10.3495	10.5779	11.0396		
	MeanBDay3	1			10.7232	10.8838	11.1990		
		2			10.8836	11.0421	11.0952		
	MeanBDay4	1			9.9978	10.1228	11.1148		
		2			9.5563	9.9895	10.6136		

ANOVA

		Sum of Squares	df	Mean Square	F	Sig.
MeanBDay1	Between Groups	.000	1	.000	.001	.979
	Within Groups	6.194	10	.619		
	Total	6.195	11			
MeanBDay2	Between Groups	.001	1	.001	.002	.963
	Within Groups	2.385	10	.238		
	Total	2.386	11			
MeanBDay3	Between Groups	.001	1	.001	.009	.927
	Within Groups	1.638	10	.164		
	Total	1.639	11			
MeanBDay4	Between Groups	.218	1	.218	.343	.571
	Within Groups	6.354	10	.635		
	Total	6.573	11			

Figure S2.

In addition, under ambient air conditions no differences were found in MRSA biofilm growth (LogCFU/mL) between day1-day2-day3-day4 post extubation, see below:

NPar Tests

Friedman Test

Ranks

	Mean Rank
MeanBDay1	2.00
MeanBDay2	2.67
MeanBDay3	3.17
MeanBDay4	2.17

Test Statistics[a]

N	6
Chi-Square	3.000
df	3
Asymp. Sig.	.392

a. Friedman Test

Figure S3.

Under ambient air+CO_2 conditions no differences were found in MRSA biofilm growth (LogCFU/mL) between day1-day2-day3-day4 post extubation, see below:

NPar Tests

Friedman Test

Ranks

	Mean Rank
Day 1CFU(B)	2.60
Day 2CFU(B)	3.00
Day 3CFU(B)	3.00
Day 4CFU(B)	1.40

Test Statistics[a]

N	5
Chi-Square	5.160
df	3
Asymp. Sig.	.160

a. Friedman Test

Figure S4. Gene expression comparing vancomycin versus control groups under Atmospheric conditions neither Atmospheric conditions $+CO_2$.

IcaC-O2 comparing vanco vs. control

Test Statistics[a]

	D1Mean	D2Mean	D3Mean	D4Mean
Mann-Whitney U	4.000	2.000	2.000	4.000
Wilcoxon W	10.000	8.000	8.000	10.000
Z	-.218	-1.091	-1.091	-.218
Asymp. Sig. (2-tailed)	.827	.275	.275	.827
Exact Sig. [2*(1-tailed Sig.)]	1.000[b]	.400[b]	.400[b]	1.000[b]

a. Grouping Variable: treatrecode

b. Not corrected for ties.

clb-O2 comparing vanco vs. control

Test Statistics[a]

	D1Mean	D2Mean	D3Mean	D4Mean
Mann-Whitney U	4.000	3.000	3.000	3.000
Wilcoxon W	10.000	9.000	9.000	9.000
Z	-.218	-.655	-.655	-.655
Asymp. Sig. (2-tailed)	.827	.513	.513	.513
Exact Sig. [2*(1-tailed Sig.)]	1.000[b]	.700[b]	.700[b]	.700[b]

a. Grouping Variable: treatrecode

b. Not corrected for ties.

ebps-O2 comparing vanco vs. control

Test Statistics[a]

	D1Mean	D2Mean	D3Mean	D4Mean
Mann-Whitney U	3.000	3.000	.000	3.000
Wilcoxon W	9.000	9.000	6.000	9.000
Z	-.655	-.655	-1.964	-.655
Asymp. Sig. (2-tailed)	.513	.513	.050	.513
Exact Sig. [2*(1-tailed Sig.)]	.700[b]	.700[b]	.100[b]	.700[b]

a. Grouping Variable: treatrecode

b. Not corrected for ties.

IcaC-CO2 comparing vanco vs. control

Test Statistics[a]

	D1Mean	D2Mean	D3Mean	D4Mean
Mann-Whitney U	3.000	1.000	3.000	3.000
Wilcoxon W	9.000	7.000	9.000	9.000
Z	-.655	-1.528	-.655	-.655
Asymp. Sig. (2-tailed)	.513	.127	.513	.513
Exact Sig. [2*(1-tailed Sig.)]	.700[b]	.200[b]	.700[b]	.700[b]

a. Grouping Variable: SysTreat_recode

b. Not corrected for ties.

clb-CO2 comparing vanco vs. control

Test Statistics[a]

	D1Mean	D2Mean	D3Mean	D4Mean
Mann-Whitney U	3.000	2.000	3.000	4.000
Wilcoxon W	9.000	8.000	9.000	10.000
Z	-.655	-1.091	-.655	-.218
Asymp. Sig. (2-tailed)	.513	.275	.513	.827
Exact Sig. [2*(1-tailed Sig.)]	.700[b]	.400[b]	.700[b]	1.000[b]

a. Grouping Variable: SysTreat_recode

b. Not corrected for ties.

ebps-CO2 comparing vanco vs. control

Test Statistics[a]

	D1Mean	D2Mean	D3Mean	D4Mean
Mann-Whitney U	2.000	1.500	.000	1.000
Wilcoxon W	8.000	7.500	6.000	7.000
Z	-1.091	-1.328	-1.964	-1.550
Asymp. Sig. (2-tailed)	.275	.184	.050	.121
Exact Sig. [2*(1-tailed Sig.)]	.400[b]	.200[b]	.100[b]	.200[b]

a. Grouping Variable: SysTreat_recode

b. Not corrected for ties.

fnb-O2 comparing vanco vs. control

Test Statistics[a]

	D1Mean	D2Mean	D3Mean	D4Mean
Mann-Whitney U	3.000	4.000	2.000	4.000
Wilcoxon W	9.000	10.000	8.000	10.000
Z	-.655	-.218	-1.091	-.218
Asymp. Sig. (2-tailed)	.513	.827	.275	.827
Exact Sig. [2*(1-tailed Sig.)]	.700[b]	1.000[b]	.400[b]	1.000[b]

a. Grouping Variable: treatrecode

b. Not corrected for ties.

fnb-CO2 comparing vanco vs. control

Test Statistics[a]

	D1Mean	D2Mean	D3Mean	D4Mean
Mann-Whitney U	3.000	4.000	2.000	3.000
Wilcoxon W	9.000	10.000	8.000	9.000
Z	-.655	-.218	-1.091	-.655
Asymp. Sig. (2-tailed)	.513	.827	.275	.513
Exact Sig. [2*(1-tailed Sig.)]	.700[b]	1.000[b]	.400[b]	.700[b]

a. Grouping Variable: SysTreat_recode

b. Not corrected for ties.

RNAIII-O2 comparing vanco vs. control

Test Statistics[a]

	D1Mean	D2Mean	D3Mean	D4Mean
Mann-Whitney U	3.000	4.000	3.000	4.000
Wilcoxon W	9.000	10.000	9.000	10.000
Z	-.655	-.218	-.655	-.218
Asymp. Sig. (2-tailed)	.513	.827	.513	.827
Exact Sig. [2*(1-tailed Sig.)]	.700[b]	1.000[b]	.700[b]	1.000[b]

a. Grouping Variable: treatrecode

b. Not corrected for ties.

RNAIII-CO2 comparing vanco vs. control

Test Statistics[a]

	D1Mean	D2Mean	D3Mean	D4Mean
Mann-Whitney U	2.000	3.000	3.000	3.000
Wilcoxon W	8.000	9.000	9.000	6.000
Z	-1.091	-.655	-.655	.000
Asymp. Sig. (2-tailed)	.275	.513	.513	1.000
Exact Sig. [2*(1-tailed Sig.)]	.400[b]	.700[b]	.700[b]	1.000[b]

a. Grouping Variable: SysTreat_recode

b. Not corrected for ties.
